# Mini-open Muscle Resection Procedure under Local Anesthesia for Lateral and Medial Epicondylitis

**DOI:** 10.4055/cios.2009.1.3.123

**Published:** 2009-08-17

**Authors:** Byung-Ki Cho, Yong-Min Kim, Dong-Soo Kim, Eui-Sung Choi, Hyun-Chul Shon, Kyoung-Jin Park, Eun-Myung Lee

**Affiliations:** Department of Orthopedic Surgery, Chungbuk National University College of Medicine, Cheongju, Korea.

**Keywords:** Lateral epicondylitis, Medial epicondylitis, Local anesthesia, Mini-open, Muscle resection

## Abstract

**Background:**

This study examined the clinical results of surgical treatment using a mini-open muscle resection procedure under local anesthesia for intractable lateral or medial epicondylitis.

**Methods:**

Forty two elbows (41 patients) were treated surgically for lateral or medial epicondylitis. The indication for surgery was refractory pain after six months of conservative treatment, or a history of more than three local injections of steroid, or severe functional impairment in the occupational activities. The treatment results were assessed in terms of the pain using the visual analogue scale (VAS), Roles & Maudsley score, and Nirschl & Pettrone grade.

**Results:**

The preoperative VAS scores of pain were an average of 5.36 at rest, 6.44 at daily activities, and 8.2 at sports or occupational activities. After surgery, the VAS scores improved significantly (*p* < 0.01): 0.3 at rest, 1.46 at daily activities, and 2.21 at sports or occupational activities. The preoperative Roles & Maudsley score was acceptable in 6 cases, and poor in 36 cases, which was changed to excellent in 23 cases, good in 16 cases, acceptable in 3 cases after surgery. According to the grading system by Nirschl & Pettrone, 23 cases were excellent, 18 cases were good, and the remaining 1 case was fair. Overall, 41 cases (97.6%) achieved satisfactory results. Postoperative complications were encountered in three cases. Subcutaneous seroma due to the leakage of joint fluid in two patients was managed by additional surgery and suction drainage, and resulted in a satisfactory outcome. One patient complained of continuous pain on occupational activity, but her pain at rest was improved greatly.

**Conclusions:**

The mini-open muscle resection procedure under local anesthesia appears to be one of effective methods for intractable lateral or medial epicondylitis.

Lateral epicondylitis (tennis elbow) and medial epicondylitis (golfer's elbow) are the most common painful syndromes resulting from overuse of the elbow. Mechanical overload and repetitive stress on a tendon with a degenerative lesion are known to be the primary causes. Conservative measures using anti-inflammatory drugs, physical therapy, and local steroid injections can be the preferred options in the early stages. However, they produce unsatisfactory outcomes that can lead to chronicity and a pervasiveness of the symptoms in many cases. In addition, considering that they are work-related disorders, the patients cannot avoid uncomfortable experiences at work due to pain and low work capacity. Therefore, a surgical approach is indicated for the treatment of lateral and medial epicondylitis in patients unresponsive to long-term conservative treatment. The currently available surgical options are open, percutaneous, and arthroscopic. Each of these options is described as a successful procedure but the comparative benefits of these approaches have yet to be determined. Although Shin et al.1) and Cho et al.2) reported the clinical outcomes of open surgery, there have been few domestic studies addressing the surgical treatment outcome compared to foreign studies. Moreover, these studies failed to provide information on the anesthesia, hospital stay, and size of the skin incision. In addition, there are very few reports on a mini-open muscle resection procedure under local anesthesia.

This study evaluated the surgical outcome and efficacy of a mini-open muscle resection procedure under local anesthesia for lateral and medial epicondylitis in patients unresponsive to conservative treatment.

## METHODS

### Materials

The study population consisted of 41 patients (42 cases) who were treated with a mini-open muscle resection procedure under local anesthesia for lateral and medial epicondylitis at our hospital between February 2003 and June 2007. There were 28 females (29 elbows) and 13 males (13 elbows). The mean age was 47.5 years (range, 38 to 61) and more than 50% of patients were aged between 40 - 49 years. Twenty-eight were right elbows and 14 were left elbows. The symptomatic elbow was on the dominant side except for 4 cases. There were 32 and 10 cases of lateral and medial epicondylitis. All patients were unilaterally affected except for 1 patient. When the patients were divided according to occupation, 17 were housewives, 21 were laborers, 2 were involved in sports activities, and 2 were office workers. The mean duration of morbidity was 28 months (range, 8 to 10 years). An average of 4.2 (range, 2 to 10) local steroid injections were administered before surgery. The mean postoperative follow-up period was 13.4 months (range, 12 to 25 months).

The indications for surgery were as follows: more than 6 months of persistent symptoms despite the aggressive conservative treatments, such as rest, drug therapy, splinting, physiotherapy, and a history of more than 3 steroid injections for treatment, and functional impairment at work and home.

### Surgical Technique

All procedures were performed by a single surgeon (Y.M.K.) using a mini-open muscle resection procedure under local anesthesia, which is a modified version of the procedure reported by Nirschl.3)

**Fig. 1:**
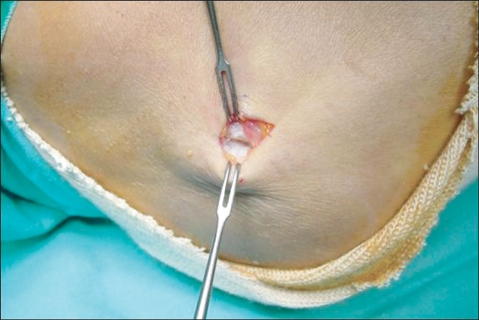
Via minimal skin incision, origin of extensor carpi radialis brevis (ECRB) muscle was approached. The origin of ECRB muscle was degenerated with partial tearing, scarring and friable granulation.

**Fig. 2:**
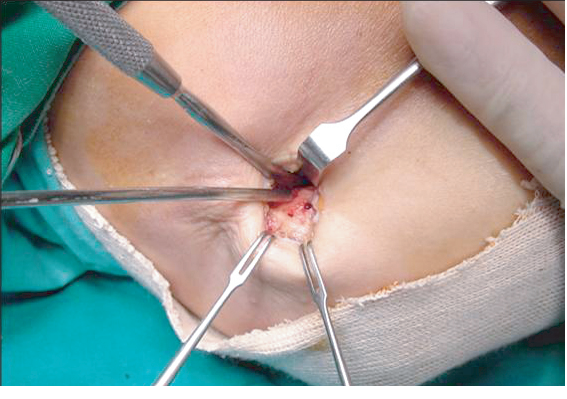
After detaching the origin of extensor carpi radialis brevis muscle, multiple holes were made by microfracture technique to ensure bleeding from the bony bed.

For lateral epicondylitis, the site of maximum tenderness over the lateral epicondyle was marked before, and an approximately 1.5 cm long skin incision was made under local anesthesia. The subcutaneous fat was held aside and the intermuscular septum was incised to allow palpation of the tip of the lateral epicondyle. The origin of the extensor carpi radialis brevis (ECRB), approximately 1cm anterior and distal to the tip, was detached from the bone using Bovie electrocautery (Fig. 1). The degenerative tendon tissue was observed at the origin of the ECRB and excised in a triangular manner. To enhance fibrous tissue regeneration at the excision site, 4-5 holes to allow blood flow were drilled in the exposed cortical bone of the lateral epicondyle using an osteotome (Fig. 2). Finally, the fascia of the ECRB was sutured, and the subcutaneous layer and skin were closed.

For medial epicondylitis, the degenerative tissue at the origin of the common flexor was exposed and removed in a similar manner. The fascia of the common flexor was closed after multiple microfracturing of the cortical bone of the medial epicondyle.

The elbow was immobilized in a long arm splint after surgery and joint movement within the comfortable range was allowed from the 1st postoperative day. Normal daily activities were allowed according to the level of pain. Progressive muscle strengthening exercises and sporting activities were allowed from the 3rd postoperative month.

### Clinical Evaluation

The pain was assessed preoperatively and postoperatively using a visual analogue scale (VAS: 0 = no pain, 10 = unbearable pain) at rest, during daily activities, and during sports or work. The preoperative and postoperative VAS scores were compared to determine the improvement in pain.

The Roles and Maudsley score and the Nirschl and Pettrone's grading system4) were used for the postoperative clinical assessment. According to the Roles and Maudsley score, the level of pain, joint movement, and activity were rated by four grades (excellent, good, acceptable, and poor). According to the Nirschl and Pettrone's grading system, excellent was defined as a full return to all activities without pain, good as a full return to all activities with occasional mild pain, fair as normal activity without pain, significant pain with heavy activity but an overall improvement in pain, and failure as no improvement in symptoms. Excellent and good results were considered satisfactory.

Statistical analysis was performed using paired t-test on SPSS ver. 10.0. A *p*-value < 0.05 was considered significant.

## RESULTS

The mean VAS score for pain at rest improved from 5.36 (range, 3 to 7) preoperatively to 0.3 (Range, 0 to 2) at the last follow-up (*p* = 0.007). The mean VAS score during daily activities improved from 6.44 (range, 3 to 8) preoperatively to 1.46 (range, 0 to 3) at the last follow-up (*p* = 0.002). The score during sports or work improved from 8.2 (range, 5 to 10) preoperatively to 2.21 (range, 0 to 5) at the last follow-up (*p* = 0.004). Overall, the level of pain improved significantly at rest, during daily activities and during sports or work (Table 1).

According to the Roles and Maudsley score, 6 and 36 cases showed acceptable and poor outcomes, respectively, before surgery. At the last follow-up, 23, 16, 3, and 0 were assessed as excellent, good, acceptable, and poor, respectively (Table 2).

According to the Nirschl and Pettrone's grading system, 41 (97.6%) of the 42 cases had satisfactory results: 23 (54.7%) cases were excellent, 18 (42.9%) were good and 1 (2.4%) was fair (Table 3). With the exclusion of one patient with a fair result, most patients showed remarkable improvement in pain and could return to their original occupation within 2-3 months after surgery.

The postoperative complications encountered included two cases of subcutaneous seroma due to the leakage of joint fluid and one case of continuous pain. The former two cases, which were noticed after the skin suture was removed, were managed with revision under local anesthesia. The leaked fluid was wiped off and a suction drainage was inserted. Continuous drainage after reclosure of the fascia led to successful healing. Continuous postoperative pain was reported by a patient in whom the preoperative pain had been too severe to raise her arm. Although the pain improved after surgery, she informed us that a return to her former occupation (carrier) was not possible.

## DISCUSSION

Lateral and medial epicondylitis are syndromes that are characterized by local pain and tenderness over the elbow, which is the origin of the tendons that move the wrist and fingers. They are associated with housework, occupation and sporting activities, and cause great discomfort during daily activities. The cause of these syndromes is unclear but some suspected causes^3^,^5^-7) are multiple ruptures at the origin of a muscle, bursitis, ossification at the origin of a muscle, synovial fold in the radiohumeral joint, entrapment of the radial nerve, and degeneration of the annular ligament. Many surgical approaches have been introduced to deal with these causes.

With regard to the pathophysiology of epicondylitis, Cyriax8) reported that tendinous changes occurred after the pathological healing of microscopic tears caused by repetitive and severe overuse, and Nirschl3) described it as ultimate ruptures of the tissues already affected by a circulation disturbance, nutritional changes, and overuse. It is believed that the overuse of degenerative tissues results in ruptures, as Nirschl3) stated, and that the high morbidity among housewives in their 40s and 50s can be explained by this theory

Lateral and medial epicondylitis are often treated with conservative measures, such as rest, medication, immobilization, physical therapy, and local steroid injection. Unfortunately, they recur in 12-30% of patients,^5^-7) and surgical procedures should be considered for failed conservative treatment in 3-8% of patients.^3^,^4^,^6^,7) It is believed that patient selection and surgical techniques are two important elements for satisfactory outcomes. In this study, the indications for surgery included the disease duration, the number of local steroid injections, and the level of functional impairment.

Lateral epicondylitis has many possible causes and a number surgical treatment options. Among them, open ECRB release, percutaneous extensor tenotomy and arthroscopic ECRB release are currently the most preferred procedures with an 80-97% success rate.^9^,10) It is unclear which procedure is best. The limitations of open ECRB release include late return to work and sporting activities due to a prolongation of the postoperative recovery time, a risk of posterolateral instability of the elbow due to lateral ligament complex injuries, and the formation of neuroma after surgery.11) Percutaneous extensor tenotomy can be effective in dealing with the downsides of open ECRB. However, it also increases the risk of recurrence due to the incomplete removal of a lesion and disrupts the concomitant treatment of an intraarticular lesion because of the limited visualization of the inside of a joint. With regard to arthroscopic ECRB release, it is difficult to suture the ruptured ECRB, to avoid the risk of injuring the lateral collateral ligament during debridement, and to become comfortable with the surgical technique in a short period.12)

Good results were obtained by performing a resection of the denatured tissue after identifying the anatomical lesion at the origin of the ECRB or that of the common flexor through a minimal skin incision under local anesthesia. In addition, the procedure produced low levels of postoperative pain, a short hospital stay and rehabilitation period, and early return to daily activities. However, a randomized comparative study with a similar study population should be carried out because it was impossible to compare the three advantages of the procedure with other studies. In addition, lesions of the inside of a joint, such as a plica or synovitis, were not identifiable. Mucoid degeneration was observed and the deposition of steroid after the injection was observed during surgery in some cases.

An improvement in the understanding of the disorder and in surgical techniques has led to the use of a smaller skin incision. However, the size of the skin incision was approximately 5-6 cm or was not even mentioned in most domestic studies. In addition, there was no information on the anesthetic method. Arthroscopy is one of the most common minimally invasive procedures, and can be performed under regional anesthesia. Therefore, it is believed that this method requires only an approximately 1.5 cm incision under local anesthesia without the need for hospitalization, and will be beneficial to patients in terms of avoiding general or regional anesthesia and reducing medical expenses.

Surgical treatments often result in unsatisfactory outcomes but there are few papers describing the cause of such failures. According to Morrey,13) patients with lateral epicondylitis required revision surgery due to the improper removal of the degenerative lesion of the ECRB or insufficient decompression of the radial tunnel. Owens et al.14) reported that a concomitant intraarticular lesion was the reason for the revision. Organ et al.15) suggested an incorrect diagnosis, entrapment of the posterior interosseous nerve, and secondary benefits, such as industrial injuries disablement benefit, might require a revision.

In this study, the complications after surgery included two cases of a subcutaneous seroma due to the leakage of joint fluid and a case of pervasive pain in the elbow. The former two were treated successfully with revision. In order to prevent the occurrence of such complications, care should be taken not to damage the articular capsule during the release of the ECRB. One patient, who could not return to work (carrier) due to pervasive pain, also stated that the preoperative severe intractable pain was reduced significantly.

Verhaar et al.16) emphasized the importance of the long-term results in determining the success or failure of surgery. Most patients were able to return to work within 2-3 postoperative months with complete symptomatic improvement.

The limitation of this study was that an analysis was not made based on a comparison with other methods of anesthesia and surgical techniques, which were mostly heavier than the present method, in terms of intraoperative discomfort, postoperative pain, medical expenses, patient satisfaction, hospital stay, and rehabilitation period. Such comparisons will be performed in further studies.

In conclusion, 41 (97.6%) out of 42 elbows with medial or lateral epicondylitis, which were unresponsive to long-term conservative treatments, were managed successfully with mini-open muscle resection procedure under local anesthesia. Overall, it is believed that this procedure is an effective treatment option that reduces the time and expense required for conservative treatments, allays the patient's concerns regarding anesthesia, and promotes a rapid return to work.

## Figures and Tables

**Table 1:**
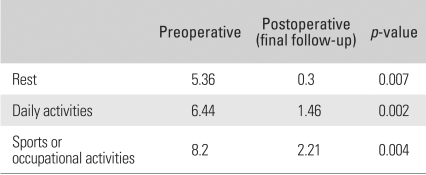
Comparison of the Pain Intensity Level Using a Visual Analogue Scale (VAS)

**Table 2:**
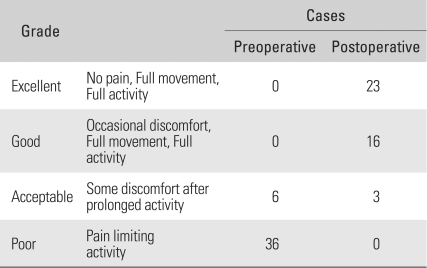
Preoperative and Postoperative Roles & Maudsley Scores

**Table 3:**
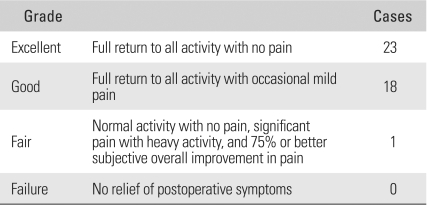
Postoperative Nirschl and Pettrone Grades
